# Prediction of Outcomes for Emergency Cervical Cerclage in the Presence of Protruding Membranes

**DOI:** 10.5402/2012/842841

**Published:** 2012-01-24

**Authors:** Purnima Deb, Nighat Aftab, Shabana Muzaffar

**Affiliations:** Department of Obstetrics and Gynaecology, Al Wasl Hospital, Dubai 9115, UAE

## Abstract

The aim of our study is to verify whether some maternal features are related to pregnancy outcomes of emergency cerclage when membranes are protruding through the dilated cervix. We present a retrospective review of 20 cases of emergency cervical cerclage performed over a 3-year period at Al Wasl hospital, a tertiary level centre in Dubai. Analysis shows presence of membrane prolapse with infection causing rupture of membranes, to be the strongest predictor of poor outcome. Analysis also reveals a significant association between initial white blood cell count and perinatal outcome. This information is helpful in decision making and counseling patients regarding the likely outcome.

## 1. Introduction

Cervical insufficiency is a difficult and confusing diagnosis. Its diagnostic criteria, etiology, and treatment are all debated. There has never been a prospective, randomized, controlled trial of cerclage versus no cerclage. Emergency or rescue cerclage as it is coined is a desperate measure to prevent fetal loss before viability of very preterm babies with poor survival.

Emergency cervical cerclage is a procedure not commonly performed in general clinical practice. The outcome of pregnancy with emergency cervical cerclage is based on limited information and success factors are not well known. Previous studies suggest that advanced cervical dilatation, significant cervical effacement, presence of prolapsed membranes, and presence of vaginal infection cause cerclage failure.

## 2. Methods

Between 2007 and 2009 in a period of three years, 20 pregnant patients with dilated cervix and protruding membranes were treated with emergency cervical cerclage at Al Wasl Hospital, Dubai. At the time of cerclage, gestational age ranged from 17 to 26 weeks (Median = 21) and the cervical dilatation was between 3 and 8 cm. In 6 women the cervix was dilated >4 cm dilated at the time of the procedure. The cervix was effaced in all patients.

Special note was made that there was no rupture of membranes or leaking of amniotic fluid vaginally. Infection was excluded clinically by absence of pyrexia and uterine tenderness, white blood cell count. High vaginal swabs and cervical swabs were taken for microbiological culture and sensitivity. Most patients did not agree for amniocentesis but were desperate to save the pregnancy.

## 3. Results

Out of 20 patients 14 patients were mildly contracting, and one of them had a large intramural fibroid in the lower segment which caused premature effacement and dilatation of the cervix. 12 of these patients received tocolytic drugs to suppress the uterine contractions. Tractocile (Atosiban) was given for at least 6–24 hours. 5 patients had positive cultures of Gardnerella vaginalis which was treated with Metronidazole; subsequent cultures were negative. Most of these patients were treated initially with broad spectrum antibiotics intravenously, to treat infection as cultures may not be available for another 48 hours. Cervical swabs and high vaginal swabs were examined for Chlamydia trachomatis (ELISA) and cultured for bacteria and mycoplasma (Bacterial vaginosis) in all patients. Blood cultures were all negative. Most of the patients were observed for 12–24 hours before insertion of the suture to ensure that all uterine contractions had settled and if infection was present subclinically. If present, it was taken care by intravenous antibiotics started at the initial assessment. Women with persisting contractions, overt infection, and those who had a recent history of antepartum hemorrhage suggestive of placental abruption, or ruptured membranes after or before admission were excluded. We waited for a period of 12–24 hours as there were two patients who ruptured their membranes and were excluded.

The patients whose uterus stopped contracting by tocolysis by infusion of tractocile (7.5 mg in 500 mL of 0.9% saline) were given NSAID (Voltaren, Diclofenac acid, 100 mg) rectally in all patients at the time of admission after assessment. Under general anesthesia they were placed in lithotomic position with steep Trendelenberg tilt, and the situation was assessed cautiously by examination with a Sims speculum. If the cervix was visible all round the bulging membrane, attempt was made to grasp the cervical lip with sponge holder gently. Then the vagina is washed with aqueous Betadine solution, and the sponge holders are brought close to each other. An attempt is made to push the membranes into the uterus with an inflated Foleys catheter with 30 mL of water, as described by Holman [1] and Sher [2]. Gentle pressure is used to replace the membranes. This allows insertion of the sutures without risk of putting a needle through the amniotic sac causing rupture of membranes and failure of continuation of the pregnancy. Amniocentesis was not attempted in any patients as we could insert the suture in all patients. We also wanted to avoid any introduction of infection and also add the risk of loss of pregnancy with amniocentesis. 18 patients in our series had Mersilene tape (Ethicon ltd) suture inserted using the McDonald technique. Four of the patients had additional sutures using interrupted nylon or prolene sutures (0 metric gauges) above the first suture or between the anterior and posterior surface of the cervix. The sutures were tied to bring the anterior and posterior surface of the cervix together, without occluding the circulation.

Tocolysis was continued for 24 hours postoperatively, and patients were observed for any pain, contractions, or other complications. Prophylactic broad spectrum antibiotics were given intravenously, before or during the procedure, and continued orally for the next 5 days. Most patients received Zinocef (Cefuroxime 1.5 gms 8 hourly) and metronidazole (1 gm 12 hourly) and were kept in hospital for 10–14 days. They were commenced on Clexane 40 mg (enoxaparine) to prevent deep vein thrombosis. Additionally, all these patients received Primolut 250 mg (progesterone) once weekly, till 32 weeks of pregnancy, which acts as a uterine relaxant. On discharge, instructions were given to lead a quiet life, avoid intercourse, and attend the antenatal clinic for assessment at 2 weeks intervals.

## 4. Results

**Figure 1:**
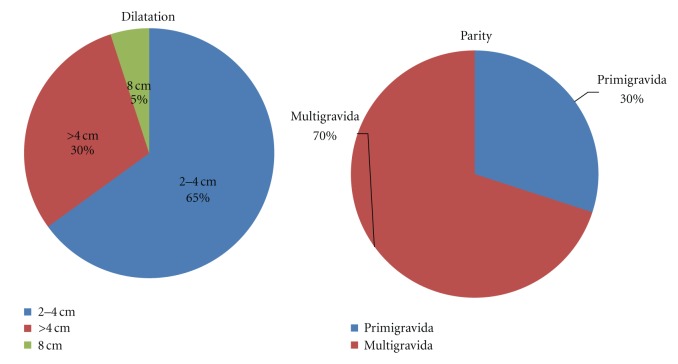


Total patients were 20. Sample Gestational age was 17–26 weeks. Most patients delivered within 4 hrs of admitting to the labor ward and all were satisfied with the outcome (Figure 1; Tables 1 and 2).

## 5. Discussions

Cervical cerclage is an intervention that is widely used to prevent miscarriage or delivery in the second trimester of pregnancy. In cases with advanced cervical dilatation and bulging membranes, it has been referred to as (heroic cerclage) or rescue cerclage due to its poor success rate [3]. We have managed a case of successful cerclage in a woman 8 cm dilated with membranes bulging up to introitus.

Mechanical support of a weak cervix was thought to be the main factor required to prolong the pregnancy. The classic description of pregnancy loss due to cervical incompetence is unexpected sudden painless delivery. Most commonly, miscarriage in the second trimester or early preterm delivery occurs following premature ripening and shortening of the cervix and the onset of painful contractions. The probable mechanism is that a degree of cervical incompetence, not sufficient to cause sudden pregnancy loss, exposes the fetal membranes to vaginal bacteria, and this leads to stimulation of the inflammatory process responsible for the onset of labor [4].

Cervical cerclage in advanced cervical dilatation with bulging membranes in the second trimester is controversial. The outcome of these pregnancies is usually poor, but without a cerclage the loss of pregnancy is inevitable. The outcome can be improved if initially a uterine contraction suppressant is used and vaginal infection can be treated [5]. These patients need a lot of counseling and be made aware of the risk of losing the pregnancy. Prolonging pregnancy to reach just viable gestations may also increase overall morbidity. It has been suggested that infection is likely to play a part in many cases of miscarriage in the second trimester and therefore screening for infection before insertion of the suture may predict prognosis. However, in women with bulging membranes, delay in the insertion of the suture is likely to increase the risk of infection, due to the increased exposure of the fetal membranes to vaginal bacteria [6, 7]. Reported survival rates following emergency cerclage vary from 12.5% to 63% [8] in women with cervical dilatation of >3 cm.

Use of tocolytics helps to suppress the uterine contractions and also reduce the intrauterine pressure thus reducing the bulging membranes. Use of NSAID drugs usually acts as anti-inflammatory and reduces the inflammatory response and reduce the liquor production.

**Figure 2:**
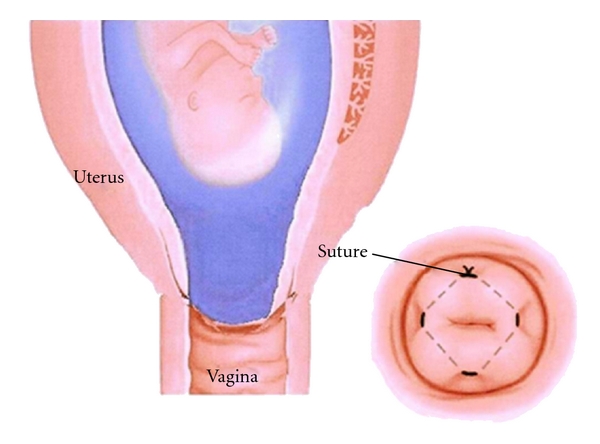


Assessment of the cervical length by transvaginal ultrasound after cervical cerclage may help predict the outcome of pregnancy [9, 10]. They showed that the length of the endocervical canal and the length of the closed cervix above the suture predicted delivery before 36 weeks of gestation. Although some authors have seen improvements in the state of the cervix, this has not been reported after cerclage with advanced cervical dilatation. Owen et al. described 29 cases of cervical cerclage following shortening or funneling of the cervix seen on transvaginal scan [11]. Cervical cerclage closes the cervix and relaxes the uterus. Our one case with advanced cervical dilatation was probably due to the presence of fibroid causing cervical dilatation, rather than infection. However, we suggest that despite an advanced degree of cervical dilatation, the insertion of a cervical suture may lead to remodeling of the cervix. By replacing the membranes and closing the cervix, the risk of exposure to vaginal infection is reduced and therefore the inflammatory-like process which is responsible for the cervical ripening and onset of contraction is also reduced. This causes the cervix to close and lengthen and prolong the pregnancy (Figure 2).

## 6. Conclusion

All women with advanced cervical effacement or dilatation or both should be counseled about the paucity of data to support the efficacy of the emergency or rescue cerclage, as well as the potential associated maternal and neonatal morbidity. Despite its overall poor prognosis, proper selection of cases results in successful outcome. Follow-up by transvaginal scan for cervical length, screen for bacterial vaginosis [12], and treatment with antibiotics covering aerobic and anaerobic organism, and pre- and postoperative tocolysis is likely to be helpful in prolonging the pregnancy and thus improving outcome. 

It is also important to exclude placental abruption and labor in women with cervical dilatation in the second trimester but we did not find this to be difficult clinically. To add, a period of observation is helpful before considering emergency cerclage. By adopting this approach there appears to be little hazard to the women and an over 60% chance of survival for the infant.

## Figures and Tables

**Table 1:** Antenatal.

Criteria	Patients	Percentage
Contractions present	14	70%
U/S assessment of cervix length (0.5–2 cm)	18	90%
Big fibroid present in lower segment (Caused cervix dilatation to 8 cm)	1	5%
Tocolysis for 6–24 hours	12	60%
WBC count >14,000	2	10%
HVS culture—Bacterial Vaginosis	5	25%
Intravenous antibiotics administered in 48 hours	20	100%
SROM within 12 hours	2	10%
Amniocentesis	0	0%
NSAID administered post-operation	18	90%
Clexane during hospital stay	18	90%
Double suture (Prolene/Nylon)	4	20%
Cervical cerclage (Mersilene tape)	18	90%
Prophylactic Broad spectrum antibiotics continued orally for 5 days	18	90%
Hospital stays for 1-2 weeks	18	90%
ANC follow up—2 weekly till delivery	20	100%
Supportive Progestational drugs—Primolut 250 mg i/m weekly till 32 weeks	18	90%

**Table 2:** Labor and delivery.

Criteria	Patients	Percentage
Ongoing pregnancy	18	90%
Cerclage removed at 36+ weeks	17	85%
Assisted vaginal delivery in 2nd stage of labor	2	10%
Delivered after their EDD	2	10%
Classical CS in previous pregnancy	1	5%
